# Porous Fluorocarbon from Rice Husk for the Efficient Separation of Gases

**DOI:** 10.1002/gch2.202000124

**Published:** 2021-05-07

**Authors:** Rashed S. Bakdash, Isam H. Aljundi, Chanbasha Basheer, Ismail Abdulazeez, Abdulaziz A. Al‐Saadi

**Affiliations:** ^1^ Department of Chemistry King Fahd University of Petroleum and Minerals Dhahran 31261 Saudi Arabia; ^2^ Department of Chemical Engineering King Fahd University of Petroleum and Minerals Dhahran 31261 Saudi Arabia

**Keywords:** fluorination, high surface area, microwave‐assisted synthesis, natural sorbent, separation factor, silica removal

## Abstract

A porous fluorocarbon sorbent is synthesized from rice husk (RH) in a microwave reactor and then evaluated for the adsorption of different gases (CH_4_, CO_2_, and N_2_). The fluorocarbon is characterized by Fourier transform infrared spectroscopy (FTIR), X‐ray diffraction (XRD), Brunauer–Emmett–Teller (BET), Raman spectroscopy, Thermal gravimetric analysis (TGA), and X‐ray photoelectron spectroscopy (XPS). Significant enhancement in the surface area of activated carbon material is obtained from 29 to 531 m^2^ g^−1^ after removing naturally present silica in RH. Results reveal that rice husk fluorocarbon (RHF) has a higher adsorption affinity for CO_2_ (1.8 mmol g^−1^) than that of the sulfonated rice husk (RHS) (1.4 mmol g^−1^) at 298 K while the corresponding separation factor of CO_2_/CH_4_ is 4 and 3; respectively. Higher separation factors of 12 and 10 are observed for the binary system of CO_2_/N_2_, respectively. Quantum chemical density functional theory (DFT) calculations agree with the experimental observations. They reveal that RHF exhibits strong columbic interactions with considerable interaction energies of −87.85, −76.75, and −55.65 kcal mol^−1^ with CO_2_, CH_4_, and N_2_ gases; respectively. Finally, the adsorption process results are highly reproducible, with a small decrease in the adsorption capacity of less than 5% after repeated trials.

## Graphical abstract

The equilibrium adsorption of different gases (CH_4_, CO_2_, and N_2_) is evaluated in fluorinated rice husk, which demonstrates higher separation factors compared to sulfonated rice husk sorbent.

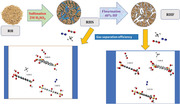

## 1 Introduction

Carbon dioxide is a greenhouse gas that is mainly found in natural gas, as a fuel‐combustion product and landfill gas. It is responsible for more than sixty percent of the greenhouse effect and its associated effects. CO_2_ emission in the kingdom of Saudi Arabia was 571 billion kg in 2018 and ranked number 13 worldwide. The state‐of‐the‐art technology for CO_2_ capture is an amine‐based absorption process, which requires substantial investment costs and high energy in addition to the instability and corrosion problems. Natural gas purification techniques to meet the pipeline quality can also include adsorption, cryogenic separation, and membrane separation. More specifically, adsorption over solid adsorbents is more effective and less expensive than liquid absorption using amine solvents.^[^ ^1^, ^2^, ^3^, ^4^, ^5^, ^6^ ^]^ Gas separation using porous solid adsorbent (especially activated carbon) is the most effective and applicable method in terms of selectivity, large‐scale of CO_2_ capture, less energy requirement, and simplicity in addition to the high thermal and chemical stability of activated carbon in acidic and basic media.^[^ ^1^, ^2^, ^3^, ^4^, ^5^, ^6^ ^]^ Using natural charcoal as nonrenewable raw materials with the long conventional process to produce activated carbons is considered as relatively expensive substances. Recently, using biological and other cheap raw materials, several studies were reported on the synthesis of carbon‐based materials with high adsorption capacity and economic impact.^[^ ^3^ ^]^ In addition, activated carbon can also be prepared from industrial wastes such as olive cake^[^ ^7^ ^]^ or sewage sludge.^[^ ^8^ ^]^ Activated carbon is now considered as a valuable material for many applications in the industry (mainly as an adsorbent for gas separation) because of its superior properties like high surface area and high adsorption capacity.

Moon et al.^[^ ^1^ ^]^ investigated the use of activated carbon in the separation of CH_4_/CO_2_ using the electric swing adsorption method. The adsorption capacity of CO_2_ was 40 mg g^−1^, and the separation factor of CH_4_/CO_2_ was two at 293 K and 1 bar. Kacem et al.^[^ ^9^ ^]^ used commercial activated carbon to study the separation of CO_2_/N_2_ and CO_2_/CH_4_ using pressure swing adsorption technique at different operating temperatures and pressures. Selectivity, adsorption capacity, and reusability were among the studied parameters of each adsorbent sample. High purity separation of binary gas mixtures of CH_4_–CO_2_ with 95% of CH_4_ in the purified gas was achieved. Shen et al.^[^ ^10^ ^]^ investigated the adsorption of N_2_ and CH_4_ at different temperatures (303–423 K) and pressure (0–100 kPa) on activated carbon beads. The adsorption capacity of CH_4_ and N_2_ was 1.9 and 0.27 mol kg^−1^, respectively, at 303 K and 100 kPa. Yi et al.,^[^ ^2^ ^]^ prepared microwave activated carbon to test the adsorption equilibrium of CH_4_, CO_2,_ and N_2_ from a mixture of gases at room temperature. The extent Langmuir and Toth's model predicted the Multi‐component adsorption system. The results revealed that the adsorption of carbon dioxide was dominant in the ternary gas mixtures with a CO_2_/CH_4_ separation factor of 4.37. Zhang et al.^[^ ^4^ ^]^ study the effectiveness of RH derived carbon materials and treated with hydrofluoric acid to adsorb CO_2_. Remarkable adsorption capacity of 77.9 mg g^−1^ at 30 °C was achieved. Álvarez‐Gutiérrez et al.^[^ ^5^ ^]^ tested the dynamic properties of CH_4_ and CO_2_ adsorption on biomass carbon‐based adsorbents in a packed‐bed setup. The CO_2_ uptake calculated from the dynamic breakthrough curves at 10 bar was found to be 5.14 mmol g^−1^ for the CS‐CO_2_ sample.

Activated carbon can be prepared from rice husk through chemical or physical activation.^[^ ^11^, ^12^, ^13^, ^14^ ^]^ Chemical activation of carbon materials can be achieved with mineral salts such as ZnCl_2_, while the physical activation is usually performed with the help of oxidizing agents like carbon dioxide or steam. For chemical activation, the precursor is blended with an activating agent, followed by carbonization by conventional heating or microwave heating.^[^ ^15^ ^]^

Microwave‐assisted heating is attractive in many applications due to its ability to shorten the reaction time and the consumed energy. Unlike the surface heating in conventional ovens, microwave‐assisted heat is both within and volumetric. Besides, a large thermal gradient is produced by microwave heating, which shortens reaction time with high effectiveness and temperatures.^[^ ^16^ ^]^

This study focused on the preparation of rice husk derived fluorocarbon (RHF) using the microwave‐assisted technique. The sorbent was investigated for the adsorption of various gases (CH_4_, CO_2_, and N_2_). First principle density functional theory (DFT) studies were performed on the bond properties and adsorption energies of CO_2_, CH_4_, and N_2_ and correlated with experimental data.

## 2 Experimental Section

### 2.1 Synthesis of Sulfonated and Fluorinated Rice Husk

Rice husk was a spinoff of rice utilized to prepare RHF. Sulfuric acid (H_2_SO_4_, 95%) and hydrofluoric acid (HF, 40%) were obtained from Sigma Aldrich (St. Louis, USA). The rice husk was washed and ground before sulfonation. 20 mL of 2 m H_2_SO_4_ was added to 5 g of rice husk and digested in a 100 mL Teflon vial with the aid of a microwave reactor at 200°C for 30 min at a high, stirring rate. The microwave temperature was increased to 200 °C and held constant at 200 °C for 30 min, then decreased the temperature to 40 °C. Later, the material was transferred to an oven and dried at 60 °C overnight, followed by grinding into a small particle size. After that, the granulated material was immersed in 20% fuming sulfuric acid (20 mL) using the microwave reactor at 200 °C for 30 min. The sample was cooling down to 40 °C, then dehydrated in the oven at 150 °C overnight. The synthesized adsorbent was labeled as sulfonated rice husk (RHS).^[^ ^17^ ^]^

1 g of RHS was soaked with hydrofluoric acid (HF) (8–40%) in microwave Teflon vial and heated for 45 min at 100 °C. They produced fine powder was rinsed with double distilled water and dehydrated in the oven at 100 °C overnight. A number of experiments were performed to obtain the optimum volume of HF added to RHS. The results showed that 8.0% was enough to eliminate the silica from RHS altogether. The resultant rice husk derived fluorocarbon was labeled as RHF.^[^ ^18^ ^]^

### 2.2 Material Characterization

#### 2.2.1 Surface Morphology

Surface morphology of RHS and RHF materials were investigated using scanning electron microscopy (SEM) (Lyra3 TESCAN). Elemental composition was determined using energy‐dispersive X‐ray spectroscopy (EDX) and X‐ray photoelectron spectrometer (V.G. Scientific ESCALAB Mk (II)) using a non‐monochromatic Al source (Kα, 1486.6 eV). The crystalline structure of the adsorbent materials as defined by the Raman spectroscopic technique (Horiba dispersive Raman spectrometer) in the wavelength range of 50–4000 cm^−1^.

#### 2.2.2 BET Surface Area

Measuring the surface area was performed on the Brunauer–Emmett–Teller (BET) instrument by taking 0.1 g of the sample in a quartz tube and degassed at 200 °C for two hours in a vacuum. Nitrogen adsorption isotherms were obtained by Quanta chrome Autosorb iQ‐MP‐C‐XR. The surface area and average pore size of RHS and RHF were measured using the BET equation and DFT method, respectively.^[^ ^19^, ^20^ ^]^

#### 2.2.3 Fourier Transform Infrared Spectroscopy (FTIR)

The FTIR spectra were acquired by employing a Nicolet 6700 FT‐IR (Thermo Electron Corporation). Potassium bromide was utilized to prepare a sample pellet, and the spectra were scanned with a resolution of 4 cm^−1^ in the range of 4000–400 cm^−1^ by the assemblage of 32 scans.

#### 2.2.4 X‐Ray Diffraction (XRD)

XRD patterns of RHS and RHF adsorbent were acquired on the Rigaku Miniflex II X‐ray diffractometer (tube output voltage 30kV) at a scan rate of 2.5° min^−1^ from 3 to 100°.

#### 2.2.5 Thermal Gravimetric Analysis (TGA)

The thermal stability of the adsorbents was obtained on SDT Q 600, TGA Instruments, New Castle, DE, by calcinating the adsorbent under the flow of nitrogen (75 mL min^−1^) up to 1000 °C with a heating rate of 10 °C min^−1^.

#### 2.2.6 Gas Adsorption

The N_2,_ CO_2_, and CH_4_ gases were adsorbed by RHS and RHF using Quanta chrome Autosorb iQ‐MP‐C‐XR. Initially, about 30–100 mg of sample was degassed for 3.5 h at 350 °C. Then, the sample was loaded for the isothermal adsorption/desorption experiments. The reversibility of the adsorption, three adsorption/desorption cycles were performed for the same sample.

#### 2.2.7 Computational Procedure

Quantum chemical DFT calculations were carried out on a simplified rice husk model, RH (comprising of coronene, C_24_H_12_^[^ ^21^, ^22^, ^23^ ^]^ and silicon oxide, Si_2_O_3_ cluster^[^ ^24^ ^]^), the RHS and RHF molecules. RHS was modeled such that hydrogen atoms at the edges of RH were substituted with sulfonic groups, whereas silica was replaced by fluoro groups in RHF. Full optimizations of geometry and vibrational frequency calculations of the adsorbents (RH, RHS, and RHF) and the gases were performed using the hybrid GGA exchange‐correlation functional of Becke^[^ ^25^ ^]^ and Perdew and Wang,^[^ ^26^ ^]^ BPW91 and the 6‐31G basis set. Total energies of the natural bonding orbitals of the adsorbents and the relative binding distances with the gases were obtained. Adsorption energies (Δ*E*_ads_) of the gases on the adsorbents were calculated using the equation

(1) $$\begin{array}{c} \Delta E_{\text{ads}}\;\;\;=\;\;-\left[E_{\text{ads}/\text{gas}\;}-\left(E_{\text{ads}}+\;E_{\text{gas}}\right)\right] \end{array}$$

where, *E*_ads/gas_, *E*_ads_, and *E*_gas_ represent the free energies of the adsorbent‐gases complex, the isolated adsorbents and the isolated gas molecules, respectively. All calculations were accomplished using the Gaussian 09 package.

## 3 Results and Discussion

### 3.1 Surface Morphology

**Figure 1:**
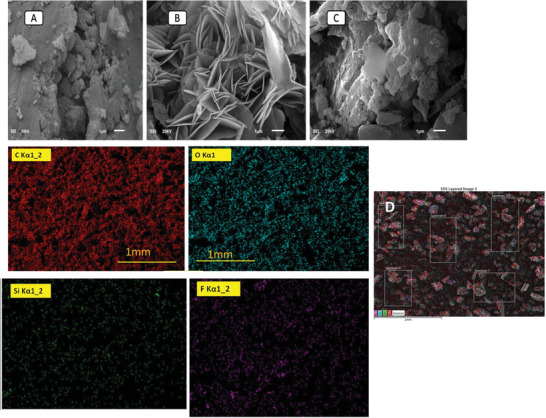
SEM micrographs of A) RH, B) RHS, C) RHF, and D) EDX elemental mapping of RHF.

Rice husk is a natural biomass material that mainly contains silica, lignin, cellulose, fat, and proteins.^[^ ^27^ ^]^ The SEM image (**Figure** **1**A) showed that small and large size silica particles occupy the side of the cell walls. The SEM image represents a smooth surface without any porous structure due to the low surface area. Results of elemental analysis from EDX of pure RH showed the presence of silicon, oxygen, carbon and nitrogen, with weight% of 18.4%, 35.2%, 43.8%, and 2.7%, respectively. The results of EDX analysis confirmed the percent of silicon in RH, closed to that reported in the literature.^[^ ^27^ ^]^ The EDX of RHS showed oxygen, silicon, and sulfur content of 44.1%, 9.1%, and 5.9%, respectively. These results indicate the functionalization of RH with sulfur after the microwave carbonization treatment with a high percentage of silica in the RHS.

From the SEM images (Figure 1B), it is clear the microwave treatment of the RH showed an ordered structure with a greater surface area. On the other hand, the surface morphology after the fluorination of RHS (Figure 1C) revealed that the hierarchical structure vanishes. The framework can be distinguished owing to the elimination of silicon. Moreover, there is a clear formation of carbon nanoparticles confirmed the presence of macropores and mesopores.

After the treatment of RHS with HF, the sulfur and oxygen contents were found to be 27.6% and 1.0%, respectively. However, the silicon content changed, and it's percent in the RHF decrease to less than 0.1%. Figure 1D shows the fluorine atoms were also found on the surface at 6.5%, which confirmed the desilication and consequent incorporation of fluorine atoms on RHF.

**Figure 2:**
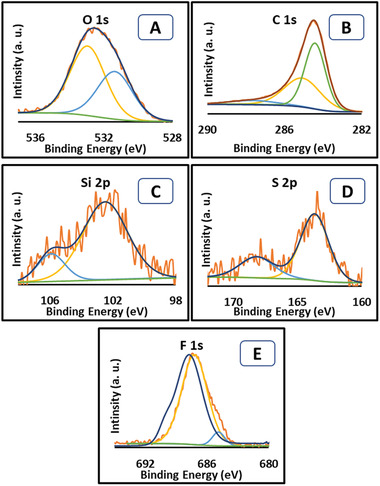
X‐ray photoelectron spectroscopy of RHF, A) wide scan spectrum, high resolution spectrum of B) O 1s, C: C 1s, D: Si 2p, E: S 2p, and F: F 1s.

High‐resolution XPS scan of RHS Figure S1, Supporting Information) revealed the presence of silicon, sulfur, oxygen and on the surface. The highest percent of oxygen in RHS is because of more elevated surface oxidation of pure rice husk material with an active oxidizing agent. The existence of oxygen and sulfur revealed the efficiency of the treatment method, which introduced oxygen‐containing —SO_3_H groups on the surface of RH material. The XPS scan of RHS revealed a band at ≈532.4 eV, which is assigned to the C=O bond of the carbonyl group.^[^ ^28^ ^]^ The binding energy present in the range of ≈162 and ≈172 eV can be attributed to S (2p) and correlated with the sulfur bonds present in —SO_4_^−2^, —SO_3_^−2^, or R—SO_3_H groups.^[^ ^29^, ^30^ ^]^ The peak at ≈168 eV was explicitly associated with the S (2p) of the —SO_3_H functional group.^[^ ^31^ ^]^ The data (**Figure** **2**D) exhibits the high‐resolution XPS band of Si (2p). The fitted binding energy peaks at ≈103.2 eV refer to the Si—O bonds.^[^ ^32^ ^]^

The high‐resolution XPS scan of RHF (Figure 2) reveals notable elemental variance in the structure. The treatment of sulfonated rice husk with HF acid led to lower in the concentration of the oxygen, sulfur and silicon with a consequent appearance of fluorine in the spectra. It was observed that there was a difference between the XPS analysis and EDX data. This can relate to the fact that XPS is a highly surface‐sensitive technique that cannot sense deeply in the material. The high‐resolution XPS data of the RHF further demonstrate the type of covalent or semi‐ionic bonding existing in fluorocarbon material. The high‐resolution spectra of carbon were decomposed into bands at ≈284.4, ≈285.0, and ≈287.7 eV, which were assigned to the sp^2^ carbon, semi‐ionic bonds, and C—F bond, respectively.^[^ ^33^ ^]^ In the case of F (1s) spectra, the band at ≈687.2 eV can be assigned to the fluorine bonded to the carbon (C—F). As the ≈687.2 eV is the dominant peak, this suggests that the plurality of the fluorine atoms in the RHF is bonded to carbon atoms with a single covalent bond.^[^ ^34^ ^]^ There is a weak band at ≈684.9 eV that can be assigned to the isolated C— F bond or C—F—C bridging bonds.^[^ ^35^ ^]^

### 3.2 Surface Area and Pore Size Distribution

**Figure 3:**
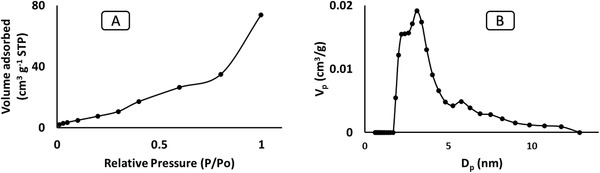
B) Pore size distribution and A) N_2_ isotherm of RH.

**Figure 4:**
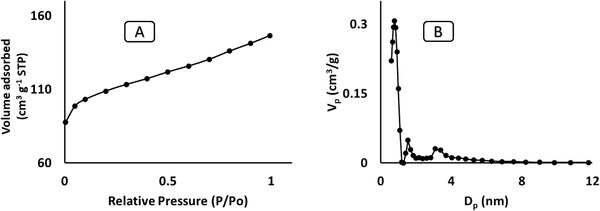
B) Pore size distribution and A) N_2_ isotherm of RHS.

**Figure 5:**
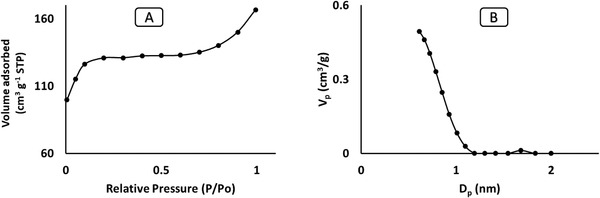
B) Pore size distribution and A) N_2_ isotherm of RHF.

Surface area and pore features of the materials were investigated using nitrogen adsorption. **Figures** **3**–**5** shows the N_2_ adsorption isotherm of RH, RHS, and RHF at 77 K. The RHF (Figure 5) demonstrates a superior adsorption capacity at relatively low pressure (P/P^o^) compared to the RHS. This could be due to the occurrence of abundant micropores in the framework of RHF. Characteristics of pure RHS and RHF, which include pore volume, pore size, and surface area, are showed in **Table** **1**.

**Table 1:** Textural properties of RH, RHS, and RHF

Sample	*S*_BET_ [m^2^ g^−1^]	*V*_p_ [cm^3^ g^−1^]	*D*_p_ [nm]
RH	29.8	0.053	3.099
RHS	410.9	0.196	0.785
RHF	531.3	0.209	0.614

The pore size distribution of RH (Figure 3) was from 3.0 to 12 nm and confirmed the presence of mesopores in pure RH that agrees with the SEM images (Figure 1A). RHS (Figure 4) consists of micropores with a diameter of 0.78 nm and mesopores with a diameter of 12 nm. RHF displayed only micropores with a pore size of 0.614 nm maximum pore size distribution of 2.0 nm in (Figure 5). The decrease in the distribution of pore size is mainly due to the sulfonation and fluorination process at high temperatures. The microporous RHF showed the highest surface area of 531 m^2^ g^−1^. The results confirm that microwave sulfonation improved the porosity of the rice husk materials. Furthermore, the treatment of sulfonated rice husk with HF acid led to the removal of the silica content and the formation of more pores with a high surface area of RHF.

### 3.3 Thermal Gravimetric Analysis (TGA)

**Figure 6:**
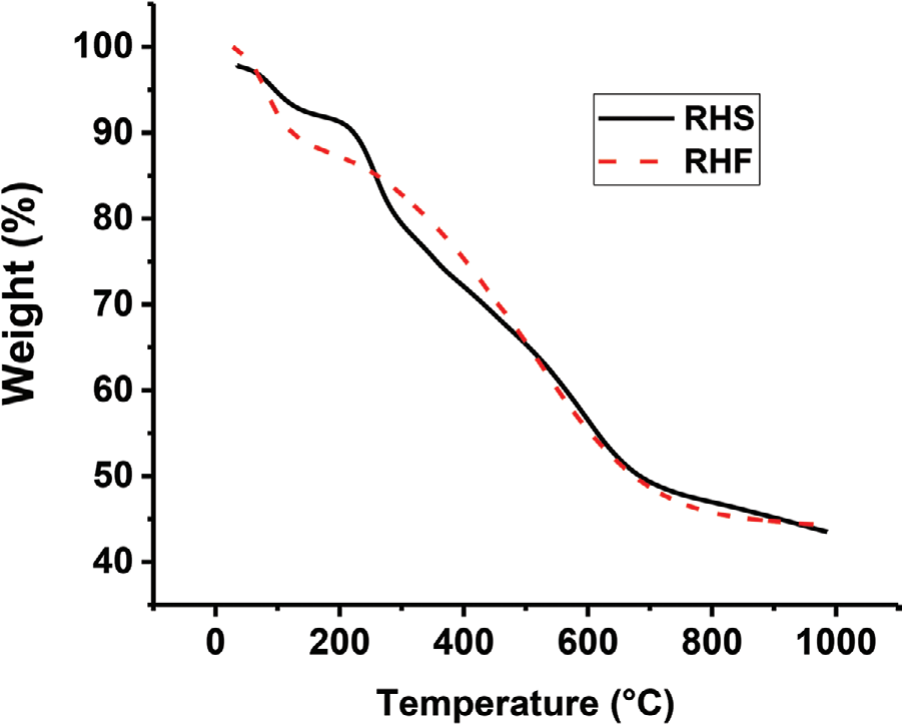
Thermo‐gravimetric analysis of RHS and RHF.

The thermal stability of the prepared materials was investigated with TGA analysis (**Figure** **6**). As the temperature reached to 1000 °C, 43.5% of RHS and 44.2% of the RHF adsorbents remained as residual solids. Mostly, three zones were shown in the TGA curves of each adsorbent. Initially, the RHS showed a decline in weight at 50–200 °C because of the removal of adsorbed moisture on the hydrophilic sulfur groups that exist on the carbon surface. In the second zone, the increase in temperature shows a noticeable decrease in the weight of RHS and RHF adsorbents, which is due to degradation of ‐COOH groups, removal of ‐OH and sulfur groups in the adsorbents.^[^ ^36^ ^]^ The third region of weight loss around 600 °C was because of the decomposition of the macromolecules and the removal of organic content.

### 3.4 Spectroscopic Characterizations

**Figure 7:**
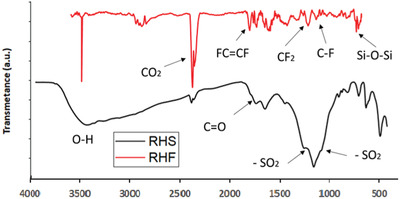
FTIR of RHS and RHF.

FTIR spectra of the RHS and RHF adsorbents are shown in **Figure** **7**, along with their characteristic absorption bands. The absorption band of sulfonated rice husk at 1036 cm^−1^ is related to ‐SO_2_ symmetrical stretching mode, while the band at 1162 cm^−1^ is associated with ‐SO_2_ nonsymmetrical stretching mode, which verified the existence of —SO_3_H group.^[^ ^37^ ^]^ The absorption band at 1620 cm^−1^ represents the polycyclic compound,^[^ ^38^ ^]^ while the stretching band at 1743 cm^−1^ represents the C=O stretching peak of the carboxylic group.^[^ ^39^ ^]^ The stretching peak of OH group is notable in sulfonated rice husk that shows the hydrophilic behavior of —SO_3_H group attached to the sulfonated rice husk surface. Furthermore, RHF displays a peak at 1082 and 1190 cm^− 1^ that confirms the existence of CF, CF_2_, and CFx groups.^[^ ^40^ ^]^ The absorption band of 749, 1726, and 1766 cm^−1^ can be accredited to the C—F bending, FC=CF stretching and F_2_C=CF bond stretching; respectively.^[^ ^41^ ^]^ The sharp peak at 600 cm^−1^ indicated the presence of silica in the RH and ascribed to the Si—O—Si bending.^[^ ^42^ ^]^ The stretching peak of OH at 3500 cm^−1^ is not shown, which confirms the less affinity of fluorocarbon to water molecules.^[^ ^33^ ^]^ RHS and RHF exhibit an absorption peak at 2355 cm^−1,^ which can be ascribed to the adsorbed CO_2_ from the atmosphere.^[^ ^43^ ^]^ Treatment of rice husk with H_2_SO_4_ resulted in an increase in porosity and hence increased the affinity of sulfonated rice husk to absorb the CO_2_ from the surrounding environment. Additional treatment of sulfonated rice husk with hydrofluoric acid to remove the silica and enhance the porosity and the surface area leads for high adsorption of CO_2_ as compared with RHS.

**Figure 8:**
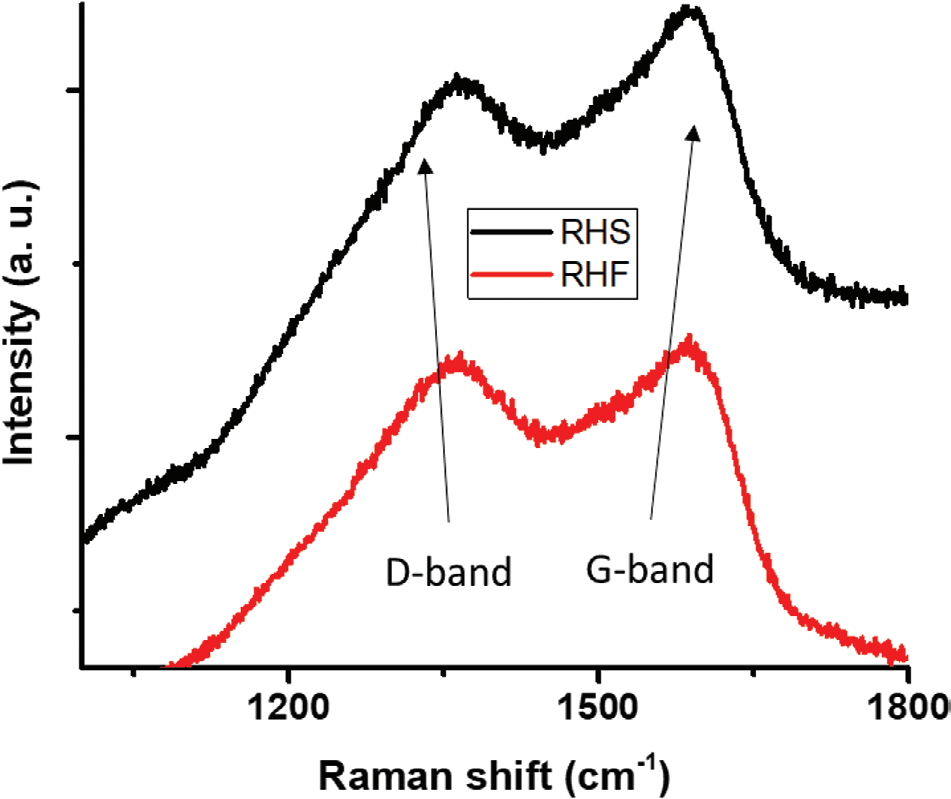
Raman shift of RHS and RHF.

Raman spectroscopy was performed to test the carbon structure derived from the RH materials. Raman spectra of RHS and RHF are showed in **Figure** **8**. The sharp peaks displayed in RHS and RHF are majorly associated with carbon vibrational modes. The Raman peak observed at 1360 cm^−1^ is related to D‐band of sp^2^ bonded carbon. The Raman peak showed at 1590 cm^−1^ is associated with G‐band due to the vibration of two carbon atoms in‐plane vibrational mode of graphite crystal sheet.^[^ ^44^ ^]^

Figure S2 in the Supporting Information displays X‐ray diffraction (XRD) patterns of RHS and RHF. A sharp peak showed around 2θ = 25^o^ with higher intensity in the case of RHS adsorbent. This peak is the attribute of aromatic carbon materials pointed in an arbitrary mode, while RHF shows a wide broad peak around 2θ = 23^o^. Relatively weak peaks between 2θ = 30–60^o^ are observed that represent the graphite structure,^[^ ^45^, ^46^ ^]^

### 3.5 Gas Adsorption Behavior

**Figure 9:**
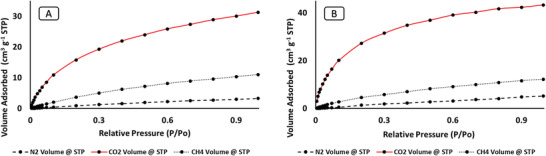
Adsorption isotherm of different gases on RHS at A) 298 and B) 273 K.

**Figure 10:**
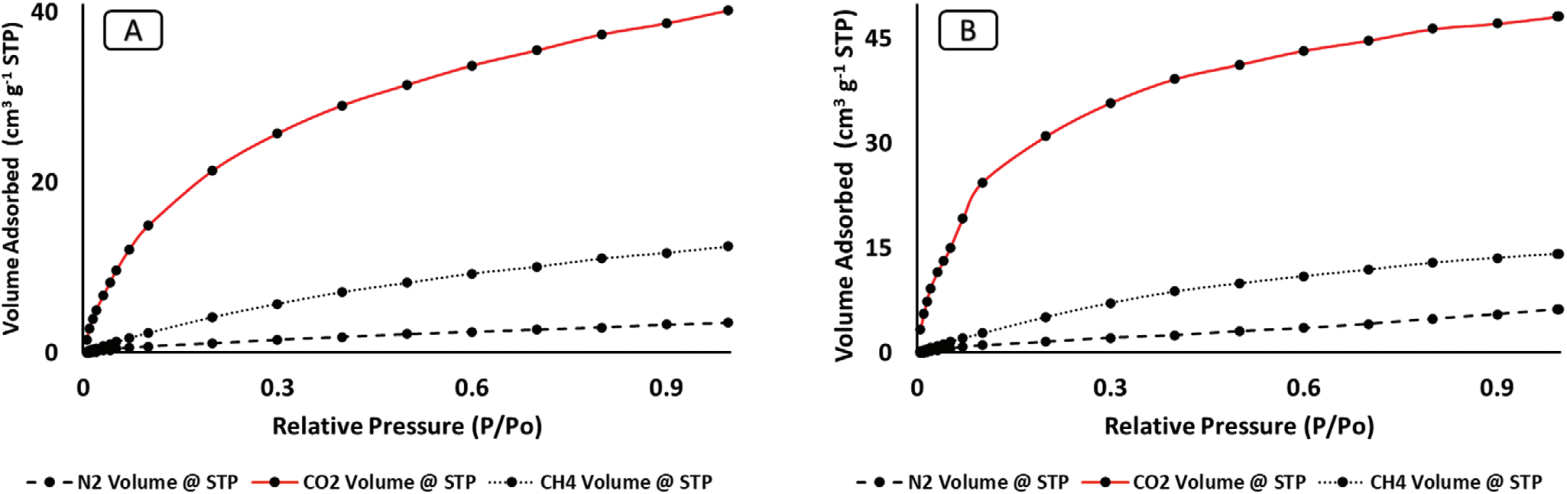
Adsorption isotherm of different gases on RHF at A) 298 and B) 273 K.

The equilibrium adsorption of CO_2_, CH_4_, and N_2_ on the prepared RHS and RHF at 273 and 298 K are shown in **Figures** **9** and **10** and Figures S3–S5 in the Supporting Information. In general, RHF showed more adsorption capacity than that of RHS due to the high surface area of RHF and the presence of the fluorine group on the surface. These results are compatible with the DFT calculations based on the binding energy and bond distance, as shown in Table S1. A decrease in the energy bandgap was observed after functionalization with the fluorine group. The adsorption capacity of nitrogen at 298 K on RHS and RHF was 0.14 and 0.16 mmol g^−1^, respectively. Furthermore, the adsorption of CH_4_ on RHS and RHF was higher than the nitrogen with an adsorption capacity of 0.49 and 0.56 mmol g^−1^ at 298 K, respectively. However, the adsorption isotherms of CO_2_ on RHS and RHF at 298 K showed more adsorption capacity of 1.4 and 1.8 mmol g^−1^, respectively. Therefore, the RHS ideal selectivity (CO_2_/CH_4_ separation factor) of 3 and 4 were obtained at 298 and 273K, respectively. The separation factor increased three times for the case of N_2_, where the ideal CO_2_/N_2_ selectivity of 9 and 10 was obtained at 298 and 273 K, respectively.

The RHF enhanced CO_2_/CH_4_ separation factor up to 4 and 12 for (CO_2_/N_2_) at 298 K. Similarly, at a lower temperature of 273 K, the separation factor of 3.5, and 8 were obtained for (CO_2_/CH_4_) and (CO_2_/N_2_) system; respectively (Figure 10). The treatment of RHS with HF acid improved the adsorption due to the following reasons (i) the reduction of silica content, (ii) the addition of fluorine functional group, (iii) increase in surface area, and (iv) changes in the porosity of the sorbent. Experimental results well aligned with the DFT calculations. Table S1 represents the affinity of RHF and RHS to the adsorption of gases. The binding energy and bond distance between the adsorbent and adsorbate plays a significant role in the adsorption capacity where the later increases in the order of CO_2_ > CH_4_ > N_2_.

**Figure 11:**
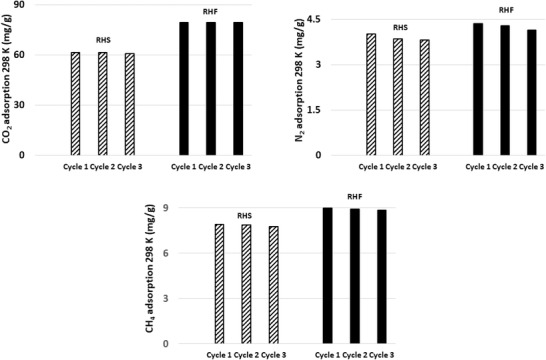
Adsorption recycle of N_2_, CH_4_, and CO_2_ on RHS and RHF at 298 K.

The reusability of RHS and RHF was investigated at 298 K for three different gases: CO_2_, CH_4_, and N_2_ (**Figure** **11**). The results showed less than a 5% decrease in the adsorption capacity between the first and third cycles, which means that these materials are highly reproducible. **Table** **2**. shows a comparison of the adsorption capacities of RHS and RHF with other carbon‐based materials reported in the literature. Comparable results are presented for N_2_, and relatively higher capacities are shown for CO_2_ and CH_4_.

**Table 2:** Comparison table of adsorption capacity of different activated carbon‐based adsorbents

Sample	Adsorption capacity of CO_2_ [mmol g^−1^]	Adsorption capacity of CH_4_ [mmol g^−1^]	Adsorption capacity of N_2_ [mmol g^−1^]	Temp. [K]	Pressure [atm]	*S*_BET_ [m^2^ g^−1^]	Ref.
*RHS	1.40	0.49	0.14	298	1	410.9	Present
*RHF	1.80	0.56	0.16	298	1	531.3	Present
AC	0.58–0.7	0.3		298	1	907–1914	(Moon and Shim, 2006)
ACF	1.5–2.1			298	1	989–2187	(Kacem et al., 2015)
ACB	1.9		0.27	303	1	845	(Shen et al., 2010)
AC	2.1	0.98	0.33	298	1	671	(Yi et al., 2013)
AC	1.1	0.7		298	1	1470	(Khalili et al., 2016)
HF‐RH	1.7			303	1	451	(Zhang et al., 2015)
CS‐CO_2_ CS‐H_2_O	1.6 1.49	0.47 0.37		303	1	1045 998	(Álvarez‐Gutiérrez et al., 2016)
PAC	2		0.2	298		1106	(Caldwell et al., 2015)
AC	1	0.8 (2 bar)		303	1	757–1178	(Peredo‐Mancilla et al., 2018)

### 3.6 Computational Results

**Figure 12:**
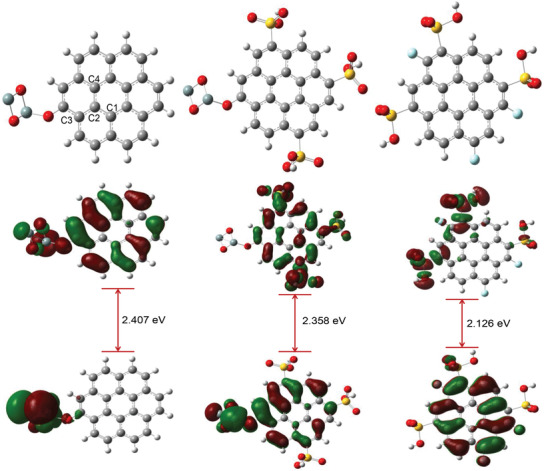
a) Optimized structures and b) frontier orbital distribution of RH, RHS, and RHF (from left to right).

Optimized molecular structures of RH, RHS, and RHF are presented in **Figure** **12**a. The charge transfer characteristics of any two interacting molecules are determined by the spatial orientation of their frontier orbitals, and the energy gap maintained therein.^[^ ^47^ ^]^ Molecules having lower energy gaps exhibits higher charge transfer characteristics and are therefore predicted to be more reactive. Frontier orbital distribution of the adsorbents (Figure 12b) showed that the HOMO‐LUMO orbitals were adequately distributed across the molecules. The RHF exhibits the lowest energy gap of 2.126 eV relative to RHS and RH with 2.358 and 2.407 eV, respectively. The functionalization of RH led to an increase in its reactivity by enhancing its charge transfer characteristics.

**Figure 13:**
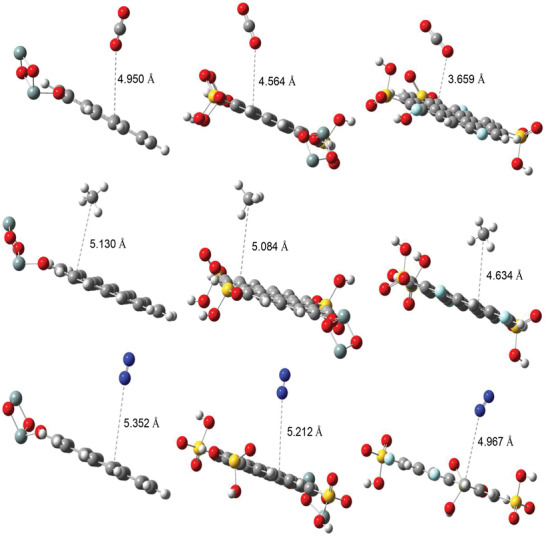
Optimized structures of a) CO_2_, b) CH_4_, and c) N_2_ adsorbed on RH, RHS, and RHF (from left to right).

Furthermore, the interaction of the adsorbents with CO_2_, CH_4_, and N_2_ gases was estimated, and the results are presented in **Figure** **13** and Table S1. Notably, the bond characteristics (bond lengths and bond angles) of the centers denoted C1, C2, C3, and C4 in Figure 13a were not significantly changed as a result of interaction with the gases for all adsorbents. However, the binding distance between the adsorbents and the gases suggested that RHF exhibits stronger interactions (shorter distances) with CO_2_ (3.659 Å), CH_4_ (4.630 Å) and N_2_ (4.967 Å). In comparison, RH and RHS are compatible with the predicted reactivity order from frontier orbital distribution. The relatively stronger interactions of RHF with the gases could be as a result of significant Columbic attractions between the adsorbent and the gases leading to physical adsorption (physisorption) as implied by the binding distances.^[^ ^48^, ^49^, ^50^ ^]^ Moreover, adsorption energies of the gases on the adsorbents further suggested that stronger interactions exist between RHF and the gases. Values of Δ*E*_ads_ of −87.65, −76.75, and −55.65 kcal mol^−1^ for CO_2_, CH_4_ and N_2_ gases, respectively relative to RH and RHS in the order RHF > RHS > RH. Overall, DFT results are in good correlation with experimental data. Functionalization of RH with sulfonic and fluoro groups led to an increase in charge transfer characteristics of the adsorbent, which consequently increases adsorption efficiency.

## 4 Conclusion

Sulfonated rice husks and fluorocarbon were successfully synthesized from rice husk associated with economic impact and environmentally friendly approach. The treatment of rice husk with sulfuric acid profoundly improved the surface area from 29 up to 410 m^2^ g^−1^. Further treatment of RHS with hydrofluoric acid increased the surface area up to 530 m^2^ g^−1^. This enhancement in the surface area is due to silica removal, which leads to improved RHF adsorption capacity of all gases compared to that of RHS. Quantum chemical DFT studies revealed that RHF exhibited higher interaction energies of −87.85, −76.75, and −55.65 kcal mol^−1^ with CO_2_, CH_4_, and N_2_ gases; respectively which was attributed to increased columbic attractions which were inconsonant with the experimental observations. The adsorption capacity of CO_2_, CH_4_, N_2_ on RHF was 1.8, 0.56, 0.16 mmol g^−1^, respectively. The recycling results revealed that the adsorption process is highly reproducible, with a reduction in the adsorption capacity of less than 5%. The separation of a mixture of gases is of great importance to the industry. Our results show that porous fluorinated carbon materials exhibit high storage capacity and separation capacity. The main challenges for efficient separation of the gas mixture are to have a higher separation factor between CO_2_/CH_4_, N_2_/CH_4_ and CO_2_/CH_4_. In this regard, further tailoring of RHF is needed to achieve a higher separation capacity that suits industrial applications.

## Conflict of Interest

The authors declare no conflict of interest.

## Supporting information

Supporting Information

## Data Availability

Data are included in the manuscript
