# Supplementary material for: Porous Fluorocarbon from Rice Husk for the Efficient Separation of Gases
Source: Glob Chall. 2021 May 7;5(7):2000124. doi: 10.1002/gch2.202000124 (PMC8272015; doi:10.1002/gch2.202000124)
Supplement: Supplementary file 1 — Supporting Information [file GCH2-5-2000124-s001.pdf]

© 2021 Wiley-VCH GmbH

Global Challenges
Open Access

Supporting Information

for *Global Challenges,* DOI: 10.1002/gch2.202000124

Porous Fluorocarbon from Rice Husk for the Efficient Separation of Gases

Rashed S. Bakdash, Isam H. Aljundi, Chanbasha Basheer,* Ismail Abdulazeez, and Abdulaziz A. Al-Saadi*

Rice husk derived fluorocarbon for the efficient adsorption of different gases

Rashed S. Bakdash[1], Isam. H. Aljundi[2*], Chanbasha Basheer[1]*, Ismail Abdulazeez[1], Abdulaziz A. Al-Saadi[1]

Supplementary materials

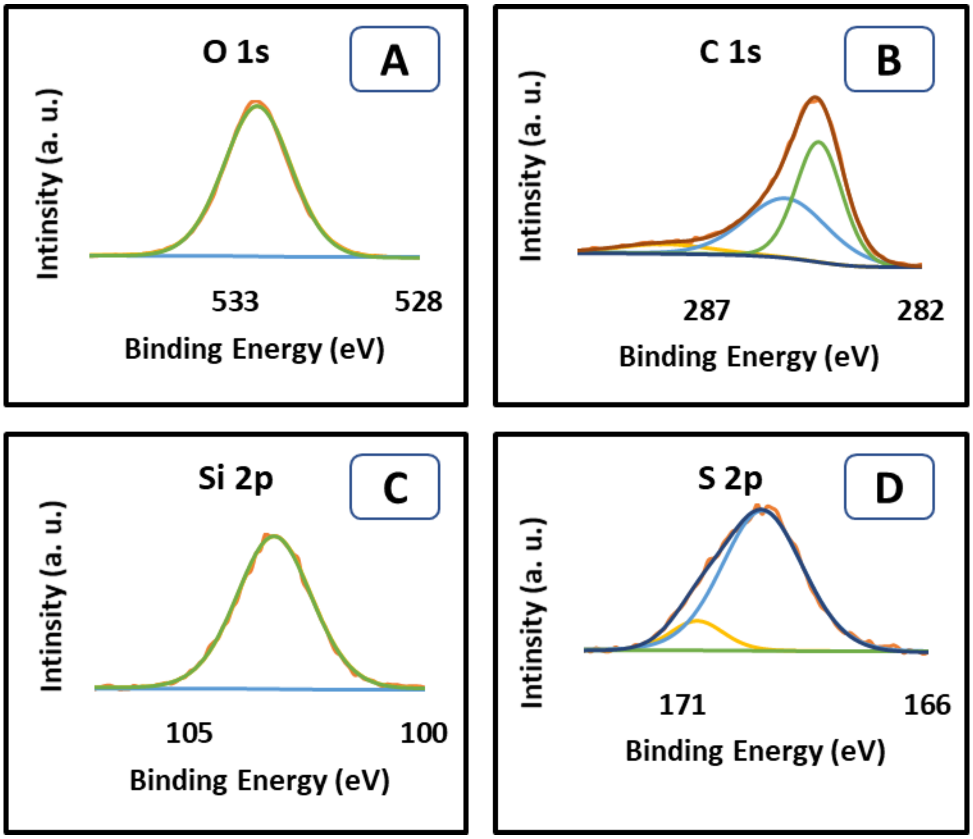

Fig. S1 X-ray photoelectron spectroscopy of RHS, high resolution spectrum of A: O 1s, B: C 1s, C: Si 2p and D: S 2p

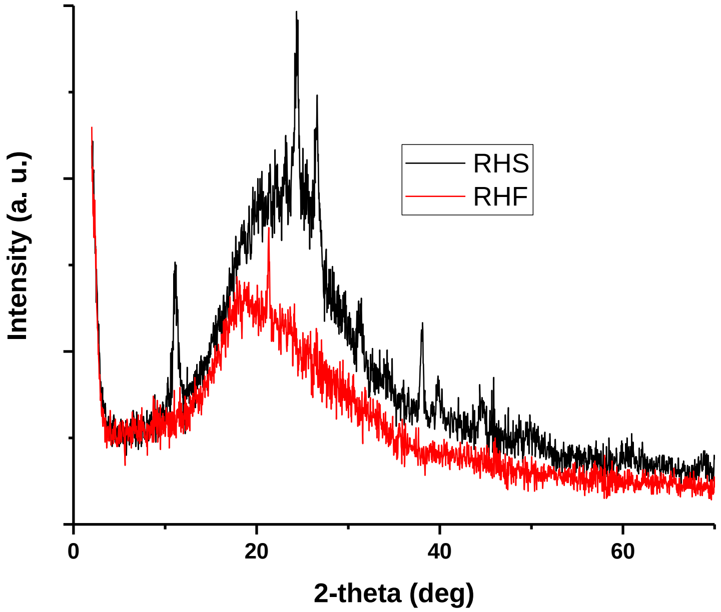

Fig. S2 X-ray diffraction patterns of RHS and RHF

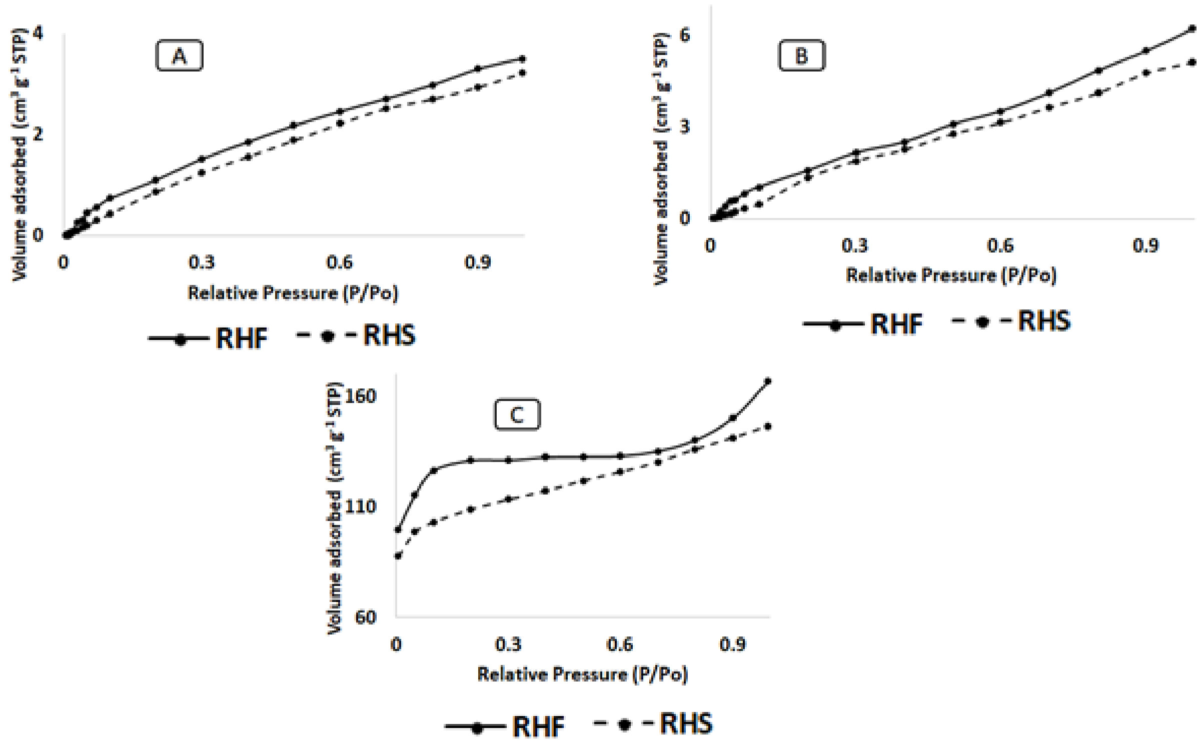

Fig. S3 N_2 adsorption isotherm of RHS and RHF at A: 298, B: 273 and C: 77 K

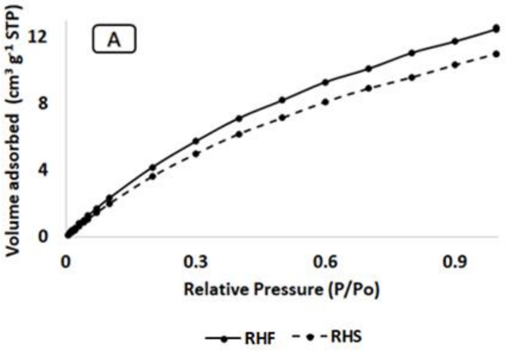

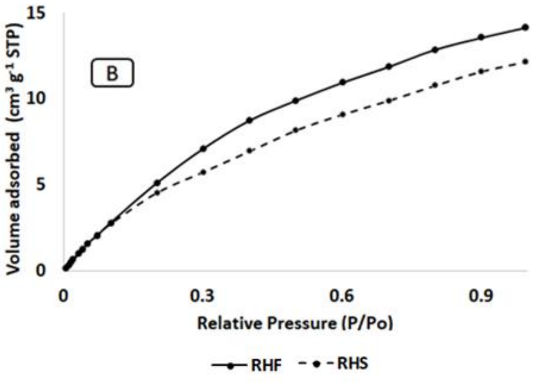

Fig. S4 CH_4 adsorption isotherm at A: 298 and B: 273 K

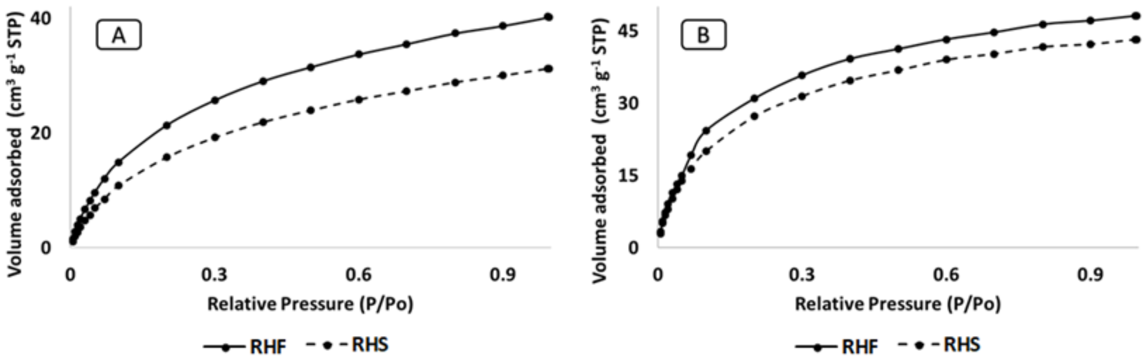

Fig. S5 CO_2 adsorption isotherm at A: 298 and B: 273 K

Table S1 Bond properties of selected atoms, binding distances and adsorption energies of CO_2, CH_4 and N_2 on RH, RHS and RHF

**Interaction**	**Bond**	**Bond distance (Å)**[a]	**Angle**	**Bond angle (°)**[b]	**Binding distance (Å)**	**Adsorption energy (kcal/mol)**
CO_2/RH	C1-C2<br>C2-C3<br>C2-C4	1.427 (1.427)<br>1.421 (1.421)<br>1.427 (1.427)	C1-C2-C3<br>C1-C2-C4<br>C3-C2-C4	120.0 (120.0)<br>119.9 (119.9)<br>119.9 (120.0)	4.950	-50.20
CO_2/RHS	C1-C2<br>C2-C3<br>C2-C4	1.429 (1.431)<br>1.418 (1.436)<br>1.428 (1.429)	C1-C2-C3<br>C1-C2-C4<br>C3-C2-C4	119.8 (119.8)<br>120.4 (119.0)<br>119.6 (121.1)	4.564	-75.30
CO_2/RHF	C1-C2<br>C2-C3<br>C2-C4	1.427 (1.428)<br>1.429 (1.428)<br>1.428 (1.427)	C1-C2-C3<br>C1-C2-C4<br>C3-C2-C4	119.8 (119.7)<br>119.3 (119.4)<br>120.7 (120.8)	3.659	-87.85
CH_4/RH	C1-C2<br>C2-C3<br>C2-C4	1.427 (1.427)<br>1.421 (1.421)<br>1.427 (1.427)	C1-C2-C3<br>C1-C2-C4<br>C3-C2-C4	120.0 (120.0)<br>119.9 (119.9)<br>119.9 (120.0)	5.130	-45.60
CH_4/RHS	C1-C2<br>C2-C3<br>C2-C4	1.428 (1.431)<br>1.427 (1.436)<br>1.429 (1.429)	C1-C2-C3<br>C1-C2-C4<br>C3-C2-C4	119.7 (119.8)<br>119.5 (119.0)<br>120.7 (121.1)	5.084	-58.25
CH_4/RHF	C1-C2<br>C2-C3<br>C2-C4	1.428 (1.428)<br>1.428 (1.428)<br>1.428 (1.427)	C1-C2-C3<br>C1-C2-C4<br>C3-C2-C4	120.0 (119.7)<br>119.9 (119.4)<br>119.7 (120.8)	4.630	-76.75
N_2/RH	C1-C2<br>C2-C3<br>C2-C4	1.427 (1.427)<br>1.421 (1.421)<br>1.427 (1.427)	C1-C2-C3<br>C1-C2-C4<br>C3-C2-C4	120.0 (120.0)<br>119.9 (119.9)<br>120.0 (120.0)	5.352	-43.20
N_2/RHS	C1-C2<br>C2-C3<br>C2-C4	1.428 (1.431)<br>1.427 (1.436)<br>1.429 (1.429)	C1-C2-C3<br>C1-C2-C4<br>C3-C2-C4	119.7 (119.8)<br>119.5 (119.0)<br>120.7 (121.1)	5.212	-46.36
N_2/RHF	C1-C2<br>C2-C3<br>C2-C4	1.428 (1.428)<br>1.428 (1.436)<br>1.427 (1.427)	C1-C2-C3<br>C1-C2-C4<br>C3-C2-C4	119.8 (119.7)<br>119.3 (119.4)<br>120.8 (120.8)	4.967	-55.65

[a] Values in parenthesis represents bond distances in isolated RH, RHS and RHF
[b] Values in parenthesis represents bond angles in isolated RH, RHS and RHF
